# Stressors impair odor recognition memory via an olfactory bulb-dependent noradrenergic mechanism

**DOI:** 10.3389/fnint.2013.00097

**Published:** 2013-12-23

**Authors:** Laura C. Manella, Samuel Alperin, Christiane Linster

**Affiliations:** Department of Neurobiology and Behavior, Cornell UniversityIthaca, NY, USA

**Keywords:** norepinephrine, noradrenaline, olfactory bulb, odor memory, stress, arousal, locus coeruleus

## Abstract

Non-associative habituation and odor recognition tasks have been widely used to probe questions of social recognition, odor memory duration, and odor memory specificity. Among others, these paradigms have provided valuable insight into how neuromodulation, and specifically norepinephrine/noradrenaline (NE) influences odor memory. In general, NE levels are modulated by arousal, stress, and behavioral state, but there is sparse evidence of a direct relationship between NE and odor memory in adult rodents. The present study uses simple mild psychological stressors (bright light and sound) to modulate NE levels physiologically in order to probe stressors NE-dependent effect on odor recognition memory. In rats with bilateral bulbar cannulations, we show that these stressors modulate olfactory memory and that this effect is at least partially mediated by the olfactory bulb. Specifically, we show that the presence of stressors during the acquisition of odor memory suppresses memory for an odor when tested 30 min after familiarization to that odor. This suppression is blocked by infusing NE antagonists into the olfactory bulb prior to odor acquisition. Additionally, we find that infusion of bulbar NE is sufficient to suppress odor memory in a manner mimicking that of our stressors. These effects are unlikely to be solely mediated by locomotor/exploratory changes produced by stressors, although these stressors influence certain behaviors not directly related to odor investigation. This study provides important information about how behaviorally relevant changes in NE can influence top-down sensory processing and odor memory.

## Introduction

Object recognition is a standard behavioral paradigm used to test non-associative recognition memory in animals. An object recognition task generally consists of two trials: a familiarization trial in which the animal has a chance to investigate an object, followed by a test trial in which the animal is presented with the familiar and a novel object. If the animal investigates the novel object more vigorously than the familiar object during the test trial, this is taken as an indication that the animal remembers the familiar object from the previous trial (Bevins and Besheer, 2006). The object recognition task can be used to test memory duration by varying the intertrial interval (ITI) between the familiarization trial and the test trial, as well as memory specificity by varying the similarity of familiar and novel objects. This task relies on the robust neophilic tendency characteristic of rodents and many other animals. This type of task has been used to test a diverse set of questions including, but not limited to the effects of stress (Beck and Luine, 1999; Bowman et al., 2003; Okuda et al., 2004; Scullion et al., 2009; Cazakoff et al., 2010), neuromodulation (Roozendaal et al., 2008; Jurado-Berbel et al., 2010), and aging and gene mutations (Bevins and Besheer, 2006) on memory.

Odor recognition tasks are similar to object recognition tasks in that they also rely on neophilia toward novel versus familiar odorants. Odor recognition and very similar habituation tasks have been successfully used to probe questions of social recognition, odor learning, memory duration, and contributions of various brain areas in these processes (Robert, 1993; Ferreira et al., 1999; Petrulis and Johnston, 1999; Johnston and Peng, 2000; McNamara et al., 2008; Wilson and Linster, 2008; Linster et al., 2009). Habituation has also been used recently using non-social odors to address questions of neuromodulation in general, and its specific effects in the olfactory bulb (OB) (Wilson and Sullivan, 1992; Wilson and Stevenson, 2003; Veyrac et al., 2007; Guerin et al., 2008; Mandairon et al., 2008; Mandairon and Linster, 2009; Escanilla et al., 2010; Freedman et al., 2013).

Non-associative odor learning has been shown to depend critically on a variety of neuromodulators. In particular, manipulation of norepinephrine/noradrenaline (NE) within the OB has pronounced effects on behavior. The OB is the recipient of rich centrifugal projections from noradrenergic neurons in the locus coeruleus (LC) (Aston-Jones et al., 2000; Sara, 2009). To date, most studies that manipulate NE within the OB show an effect on the novelty detection aspect of olfactory non-associative learning but not the memory formation *per se* (but see Guerin et al., 2008). Enhancement of NE in the OB increases spontaneous discrimination of similar odors (Escanilla et al., 2010), whereas blockade of noradrenergic α_1_ receptors in the OB impairs spontaneous discrimination of similar odors (Doucette et al., 2007; Mandairon et al., 2008). At the neural level, stimulation of the LC paired with odor presentations in anesthetized animals produced a marked reduction in mitral cell responses (the main output cells of the OB) to odor, and correlated with behavioral recognition memory to the paired odor later in the awake animal (Shea et al., 2008). Therefore, the LC stimulation-induced reduction of mitral cell activity is likely produced by a plasticity that is related to the long term odor recognition memory. A proposed explanation for behavioral recognition via anesthetized habituation in odor responsiveness of mitral cells is related to a study by Chaudhury et al. (2010). In this study, rate of habituation of mitral cell responsiveness to odors in anesthetized animals was comparable to behavioral habituation in awake animals when equating for the total amount of odor exposure and patterns of odor exposure between anesthetized and awake animals. Moreover, brain slice experiments show that NE affects excitability of both mitral and granule cells in the OB (Jiang et al., 1996; Nai et al., 2009; Smith et al., 2009). Computational models of NE modulation in the OB incorporating known physiological features can readily account for the influence of NE on processing of very low odor concentrations and odor discrimination (Escanilla et al., 2010; Linster et al., 2011; Devore and Linster, 2012).

While these studies have shed a great deal of light on the role of NE in OB odor processing, we still know relatively little about the relationship between endogenous patterns of NE release–such as those based on changes in vigilance (Rajkowski et al., 1994), arousal (Aston-Jones et al., 1996, 2000), wakefulness (Pavcovich and Ramirez, 1991; Rajkowski et al., 1994; Sands et al., 2000) or acute stress (Axelrod and Reisine, 1984; Aston-Jones et al., 1996, 2000; Valentino and Van Bockstaele, 2008)—and OB odor processing in adult rodents. However, the roles of stress, glucocorticoids, and NE have been well characterized in neonatal olfactory learning. Critical periods both overlap with and are dependent upon levels of corticosterone, stress, and specifically NE release into the OB (Moriceau and Sullivan, 2004; Moriceau et al., 2009, 2010). In adults, the application of an acute stressor that activates the LC (Valentino and Van Bockstaele, 2008), such as a bright light or sound, or a context that modifies the arousal of the animal can affect memory consolidation and recall, although the precise effects depend on the timing, context, intensity and duration of the stressor involved (reviewed in Joels et al., 2006; Sandi and Pinelo-Nava, 2007). However, these particular effects of acute stress have not been studied in the adult olfactory system.

In the present study, we examine whether natural changes in the NE levels in the OB affect short term odor recognition memory. We use a non-associative odor recognition paradigm to investigate how mild acute stressors—bright light or sound—modulate odor memory. Our results demonstrate that the delivery of a mild acute stressor during the familiarization trial of an odor recognition task suppresses odor memory. We further show that this effect is at least partially mediated by OB NE: the effects of stressors can be blocked by bulbar NE antagonists and can be mimicked by infusion of NE into the OB.

## Materials and methods

### Animals

Adult male Long Evans Hooded rats, (Charles River Laboratories, Wilmington, MA, USA) initially weighing between 250 and 300 g were used. 12 rats were used for experiment 1 (odor memory duration). A second group of 12 rats was used for experiment 2 (modulation of odor memory). Rats were housed singly in standard laboratory cages (46 × 24 cm). Animals were housed on a reversed 12-h light cycle in constant temperature and food and water available *ad libitum*. All procedures were approved by the Cornell University Institutional Animal Care and Use Committee and were in accordance with NIH guidelines.

### Cannulation surgery

Rats underwent bilateral cannulation surgery under asceptic conditions. Anesthesia was induced using an intraperitoneal injection of ketamine-xylazine (50 and 5 mg/kg, respectively) and maintained using 1–3% isoflurane. At the start of the surgical procedure, rats received subcutaneous injections of prophylactic anitibiotics (Baytril, 5 mg/kg) and analgesics (meloxicam, 2 mg/kg and butorphanol, 2 mg/kg). Rats were then affixed to a stereotaxic instrument (David Kopf Instruments, Tujunga, CA) and the skull overlying the OB s was exposed. Guide cannulae (22-gauge; Plastics One, Roanoke, VA) were implanted bilaterally into the OB s at coordinates with respect to bregma (AP: +8 mm; ML: ±1.5 mm; DV: −4.5 mm), 1 mm dorsal to the target infusion site, and affixed to the skull using skull screws (Plastics One, Roanoke, VA) and dental cement. Each infusion cannula (28-gauge, Plastics One, Roanoke, VA) extended 1 mm from the end of the guide cannula directly into the center of the OB. Dummy cannulae were placed into the guide cannulae to keep the guide cannulae free of debris and prevent infection. Rats were allowed to recover for at least five days before acclimation to the experimental procedures commenced.

### Drugs

All drugs were diluted in 0.9% sterile saline, which was also used as a vehicle control. To block NE signaling in the OB, we used a cocktail of NE antagonists consisting of the α_1_-receptor antagonist prazosin hydrochloride (1mM, Sigma, Natick, MA), the α_2_-receptor antagonist yohimbine hydrochloride (2 mM, MP Biomedicals, Solon, OH), and a non-selective β-receptor antagonist alprenolol hydrochloride (120 mM, Sigma, Natick, MA). NE was prepared at a variety of dosages (L-(−)-Norepinephrine (+)-bitartrate salt monohydrate; Sigma, Natick, MA). Drugs were prepared before beginning the experiment, separated into aliquots, and frozen at −20°C for daily use. During experiments, 6 μL of solution was infused into each OB simultaneously using a double-barreled Pump 11 Elite Nanomite Syringe Pump (Harvard Apparatus, Holliston, MA) at a rate of 2 μL/min. Drugs were infused 20 min prior to the first trial for a given session. Past studies have shown this volume and rate to be adequate to spread throughout the OB but unlikely to spread beyond the OB (Mandairon et al., 2006).

### Stressors

**Figure 1:**
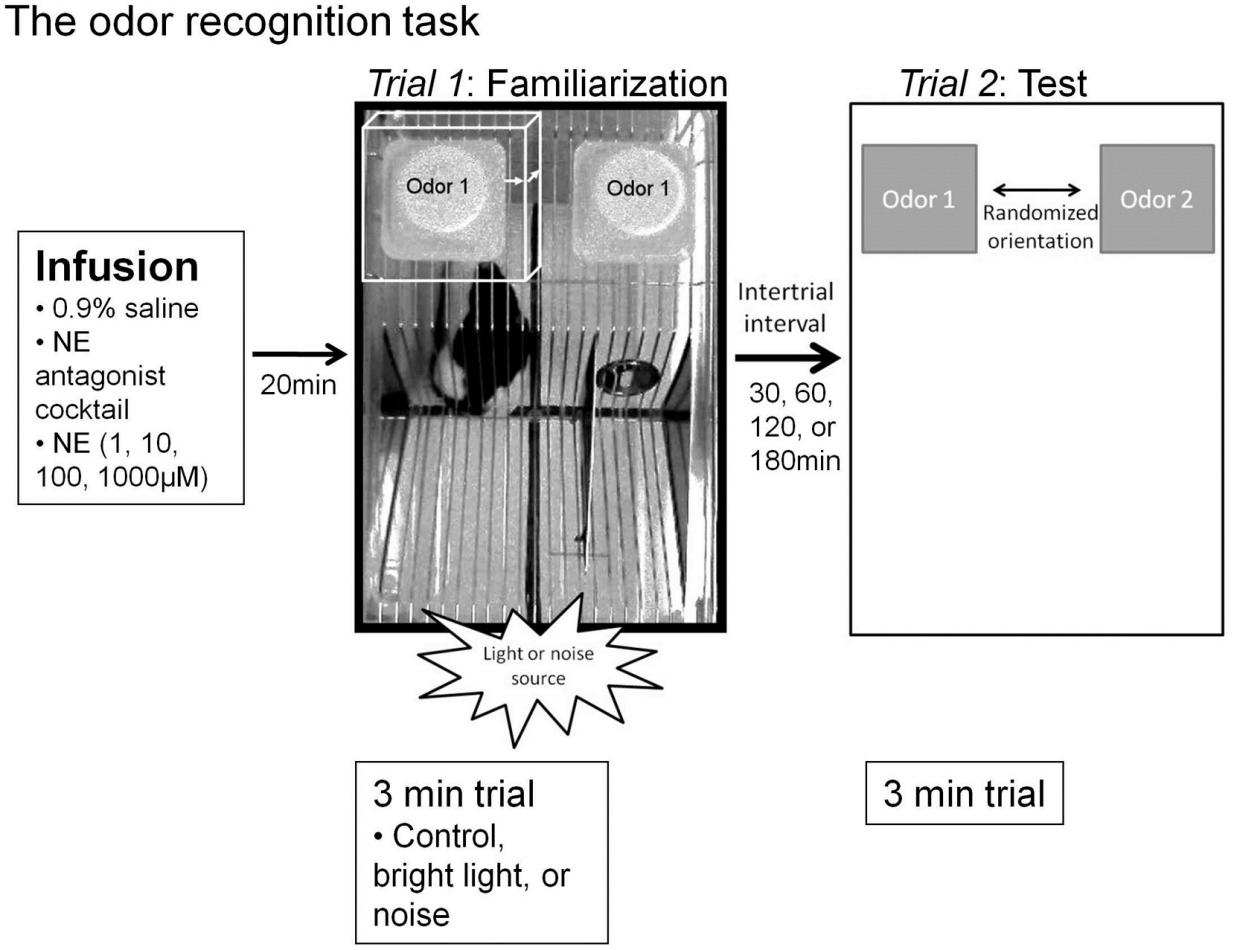
**Experimental paradigms**. The odor recognition task timeline and paradigm. Infusions were given 20 min prior to the familiarization trial (*Trial 1*) for all sessions (vehicle, NE antagonist cocktail, or a dosage of NE). For sessions where stressors were implemented, the stressor source (lamp for bright light or speaker for sound) was located adjacent to the testing chamber in the position shown in the figure during the entire odor exposure during *Trial 1*. For each trial, two inverted weigh dishes were placed on the top of the lid, in the far back corners. For *Trial 1*, as shown by an overhead view, the familiarization odor, “odor 1,” is pipetted onto a one inch square kim wipe into each of two weigh dishes. Between Trials 1 and 2, a variable intertrial interval is implemented (30, 60, 120, 180 min, depending on the particular experiment). For the *Trial 2*, odor 1 is placed in one weigh dish and a novel odor, “odor 2,” chemically and perceptually dissimilar from odor 1 is placed in a second weigh dish. The two dishes are placed in a randomized orientation. For each trial, investigation is defined as the rat's nose being within 1 cm of the weigh boat (white box in *Trial 1*, where each arrow is 1 cm). Memory is defined as significantly more investigation of odor 2 compared to odor 1. Additionally, note the quadrant markings at the base of cage in the photo of *Trial 1* that were used for transitions in video scoring.

We selected bright light and sound stimuli as mild stressors based on their history of being robust modulators of open field behavior (Archer, 1973; Roth and Katz, 1979; Katz et al., 1981) and startle response (Walker and Davis, 1997), as well as promoting phasic NE release from the LC (Koyama et al., 1994). Moreover, these are non-invasive, relatively non-traumatic stressors and pilot studies suggested that animals continue to engage in investigation of odors during familiarization trials regardless of whether a stressor was delivered. For bright light stimulation, a 40 watt desk lamp was placed at the end of the chamber facing inwards in the case of the odor recognition paradigm (Figure 1). For sound stress, we used a computer speaker playing music toward the testing chamber (Brick by Boring Brick, performed by Paramore) at a volume, on average, of approximately 70 dB, compared to background level of ambient noise of approximately 56 dB. Both the bright light and sound stressors were present for the familiarization trial (Trial1) for appropriate sessions.

### Odors

Monomolecular odorants were diluted to approximately 1 Pa vapor partial pressure to normalize the rate of particle dispersion based on concentration and volatility of odors (Table 1). For the odor recognition task, we used pairs of perceptually and chemically dissimilar odors. For a given experiment, a rat was presented with all odors sets randomly assigned across experimental conditions (Table 2).

**Table 1:** **Odor dilutions**.

**Odor name**	**% v/v Dilution**
Ethyl acetate	0.0016
Hexyl acetate	0.2274
Ethyl butyrate	0.0182
Hexyl butyrate	1.627
Hexanal	0.0222
Decanal	1.7768
Propanoic acid	0.0332
Heptanoic acid	4.6272

**Table 2:** **Odor sets**.

**Experiment 1: Memory duration**			**Experiment 2: Modulation of memory**		
**Set #**	**Odor 1**	**Odor 2**	**Set #**	**Odor 1**	**Odor 2**
1	Heptanoic acid	Hexyl butyrate	1	Propanoic acid	Ethyl acetate
2	Hexyl acetate	Decanal	2	Ethyl butyrate	Hexanal
3	Decanal	Hexyl acetate	3	Heptanoic acid	Hexyl butyrate
4	Hexyl butyrate	Heptanoic acid	4	Decanal	Hexyl acetate
			5	Ethyl acetate	Ethyl butyrate
			6	Hexanal	Ethyl acetate
			7	Hexanal	Propanoic acid
			8	Hexyl acetate	Heptanoic acid
			9	Ethyl acetate	Hexanal
			10	Hexyl butyrate	Hexyl acetate
			11	Ethyl butyrate	Propanoic acid
			12	Decanal	Hexyl butyrate
			13	Heptanoic acid	Decanal
			14	Hexyl acetate	Decanal
			15	Hexyl butyrate	Heptanoic acid
			16	Propanoic acid	Ethyl butyrate

### Odor recognition task

Rats were first acclimated to handling through at least three 10-min daily handling sessions before surgery. After surgery, rats were acclimated to infusion procedures using 0.9% sterile saline infusions and to the testing chamber and room for at least 4 sessions on separate days.

To determine the effects of bright light stress and NE within the OB on odor memory, we used an odor recognition paradigm. This paradigm consists of two three-minute trials, separated by 30, 60, 120, or 180-min intertrial intervals (ITIs). At the start of each trial the rat was transferred from its home cage into the testing chamber: a clean, clear, plastic cage (46 × 24 cm) with a wire lid (Figure 1). Following a two-minute acclimation period, odor stimuli were introduced to the rear corners of the cage lid. Odor stimuli were presented by pipetting 60 μl of diluted odors (Table 1) onto a one-inch square piece of filter paper (Kim Wipe) inside a weighing dish (Figure 1). In *Trial 1* (familiarization trial), both weigh dishes contained the same odor (referred to as the familiar odor) while in *Trial 2* (test trial) one weigh dish was scented with familiar odor and the other was scented with a novel odor (Table 2). The orientation of the two odor stimuli during the test trial was randomized (Figure 1). The amount of time spent actively investigating each odor—defined as the nose within 1 cm of the weighing dish was determined. High resolution video was recorded during all test sessions and locomotor activity of the rat was determined off-line.

### Experiment 1: odor memory duration

To determine how long rats could remember a discrete odor stimulus for use in subsequent experiments, each rat ran through a variety of ITIs between familiarization and test trials. Each rat ran through a randomized block design, experiencing one ITI per day of each of the following ITIs in a randomized order: 30, 60, 120, or 180 min. Four odor sets made up of 4 perceptually and chemically disparate odors (Table 2) were used, randomized across sessions so that each odor set had equal representation across each ITI. To be able to compare with the results from experiment 2, rats were infused with vehicle 0.9% sterile saline into their OBs bilaterally 20 min before the first trial (injection volume 6 μl, rate of 2 μl/min).

### Experiment 2: effect of stressors and bulbar NE on odor memory

Next, we determined if short-term odor recognition memory is affected by bright light or sound stressors, and NE blockers (Experiment 2a) or bulbar NE (Experiment 2b). To determine if stressors or bulbar NE infusions enhance odor memory duration, we tested ITIs of 60 and 180 min—ITIs in which our non-stressed rats could not remember, and subsequently an ITI of 30 min—an ITI in which non-stressed rats could remember (Figure 3). In order to minimize acclimation to stressors, we interleaved experimental sessions for the two experiments, randomizing all conditions across days (Table 3). Additionally, each session was spaced at least three days apart to additionally reduce acclimation to the stressors as well as to the experimental paradigm itself. For Experiment 2a and then Experiment 2b, odor sets from Table 2 were used, and odor sets were randomized across conditions, similarly to in Experiment 1.

**Table 3:** **Experiment 2 conditions (for each intertrial interval)**.

**Condition**	**Stressor**	**Drug**
1	No stress	Saline
2	No stress	NE antagonist cocktail
3	No stress	1 μM NE
4	No stress	10 μM NE
5	No stress	100 μM NE
6	No stress	1000 μM NE
7	Bright light	Saline
8	Bright light	NE antagonist cocktail
9	Sound	Saline
10	Sound	NE antagonist cocktail

#### Experiment 2a: does stress enhance odor memory (60 and 180 min ITIs)?

To determine whether stressors have an effect on odor memory and whether this effect is dependent upon OB NE, we used a 2 × 3 × 2 design where infusions of drug (saline or NE antagonist cocktail) were paired with stressors (no stress, bright light, or sound) at two ITIs (60, 180 min). As a consequence, each rat experienced 20 sessions, each time with different combinations of stress, drug and ITI (Table 3).

#### Experiment 2b: does bulbar NE enhance odor memory (60 and 180 min ITIs)?

We also determined the effect of NE infusions on odor memory during the odor recognition task. We used a 5 × 2 design, whereby each rat received each drug (vehicle 0.9% saline, 1, 10, 100 or 1000 μM NE) at different ITIs (60, 180 min), but in this case without the addition of stressors.

#### Experiments 2c and 2d: do stress or bulbar NE impair odor memory (30 min ITIs)?

After collecting the data for 60 and 180 min ITIs, we determined whether stress/NE could suppress odor memory by testing an ITI of 30 min—an ITI for which our control rats could remember. The experimental design was identical to testing the longer ITIs above, with the exception that only a 30 min ITI was tested for all conditions. Thus, each rat experienced ten sessions in a randomized order (Table 3).

### Analysis

All data analyses were done using SPSS statistical software (SPSS, Chicago, IL).

#### Odor memory

To test for odor memory duration (**Experiment 1**) and effects of NE and stressors on odor memory (**Experiment 2**), we analyzed the amount of investigation in response to familiar and novel odors during the test trial. Wilks' lambda, a multivariate analysis of variance (MANOVA) statistic, was used because MANOVA approaches to repeated-measures analyses of variance do not assume sphericity. Investigation times in response to familiar and novel odor (in seconds) were used as a within subjects factor for **Experiments 1** and **2**. For **Experiment 1**, ITI (30, 60, 120, or 180 min) was set as the between subjects factor. Results from **Experiments 2a and 2c** were analyzed using MANOVA (as above), but with stress (no stress, bright light or sound) and drug (saline or NE antagonist cocktail) as additional between subjects factors. For **Experiment 2**, analyses were run separately for each ITI tested (30, 60, and 180 min). **Experiments 2b and 2d** (NE infusions) were analyzed using MANOVA with drug dosage (saline, 1, 10, 100, or 1000 μM NE) as a between subjects factor. For each experiment (**Experiments 1, 2a-d**), *posthoc* tests (Fisher LSD, with α = 0.05) determined if rats investigated the novel odor significantly more than the familiar odor during the test trial within each experimental condition.

To further analyze **Experiment 2**, and compare the relative investigation of the novel odor across experimental conditions, we used an ANOVA with experimental group as the between subjects variable and relative investigation times as the dependent variable, followed by pairwise comparisons between experimental groups (Fisher LSD).

Data points containing outliers (more than two standard deviations from mean) were excluded from the data analysis (<10% of total). In our experiments these outliers are due to external startling stimuli or distractions.

#### Analysis of investigation time and locomotor activity during the familiarization trial

To test if differences in odor memory in **Experiment 2** could be due to variability in familiar odor investigation time during *Trial 1* (the familiarization trial), we ran an ANOVA with drug (saline, NE antagonist cocktail) or stress (no stress, light, or sound) as the between subject effects on total time spent investigating the familiarization odor as well as an ANOVA with NE dosage as the between subject effect (1, 10, 100, 1000 μM NE), both including data from all ITIs.

To test the effect of drug and stressor on investigation and locomotor activity during the odor recognition task, rats' behavior during the familiarization trials (*Trial 1*) was scored blindly by a trained observer using LabVIEW custom software. The number of rearing bouts per minute (forepaws raised from the cage floor) not including bouts when exploring the weigh boats, proportion of time spent grooming (licking the body, feet and genitals, stroking the face and whiskers with forepaws), and the rate of transitioning from quadrant to quadrants of the testing chamber (Figure 1), were measured. Data were pooled across ITIs. An ANOVA was run for each variable (rearing, grooming, and transitioning) defining drug (saline, NE antagonists) and stress (no stress, bright light, sound) as between subjects factors. To determine the effect of all drugs, an ANOVA was run for each variable, defining drug (saline, NE antagonist cocktail, 1, 10, 100, 1000 μM NE) as a between subjects factor.

### Histological verification of cannula placement

**Figure 2:**
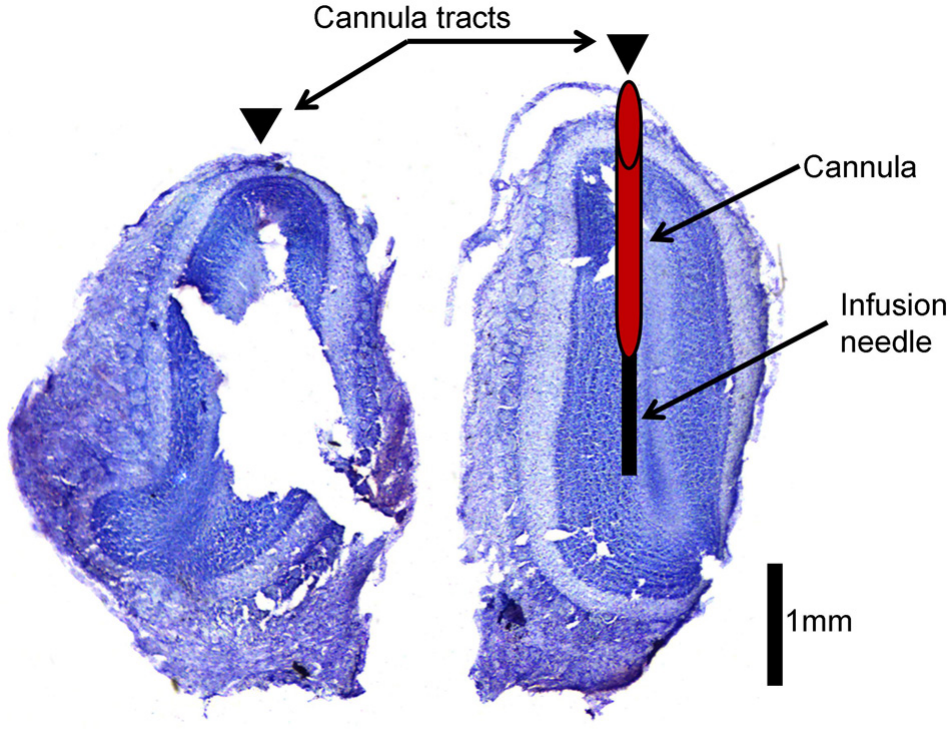
**Histological verification of cannula placement**. This is an example of an olfactory bulb slice verifying cannula placement. Arrowheads indicate location of cannula tracts. The left hemisphere indicates where cannulae were implanted (red cylinder) and how far the infusion needle extends beyond the cannula (black rectangle) during infusion of drug into the olfactory bulb.

At the end of each experiment, rats were infused with 1% methylene blue solution (in 0.9% sterile saline, 6 μl at 2 μl/min infusion rate) in order to assess the extent of diffusion within the OB of a single infusion by the beginning of a behavioral trial (Mandairon et al., 2006). After 20 min, animals were perfused transcardially using 0.9% saline followed by 10% neutral buffered formalin. Immediately following brain extraction, the OB and brain were examined visually to assess the spread of dye. Brains were stored in cryoprotectant for at least three days and then sectioned at 40 μm and stained with cresyl violet to further determine precise cannula placement within the OB (Figure 2). To view and image the slices, we used a Zeiss Stemi 2000C stereo microscope mounted on a transmitted light base with oblique illumination with dual fiber optics for reflected illumination, equipped with a Moticam 2300, 3.0 megapixel color CCD camera (Motic.com) and the Motic acquisition software.

## Results

The main goal of this study was to assess effect of mild stressors and intrabulbar infusions of NE on odor recognition memory.

### Rats remember odor objects for 30, but not 60 min (experiment 1).

**Figure 3:**
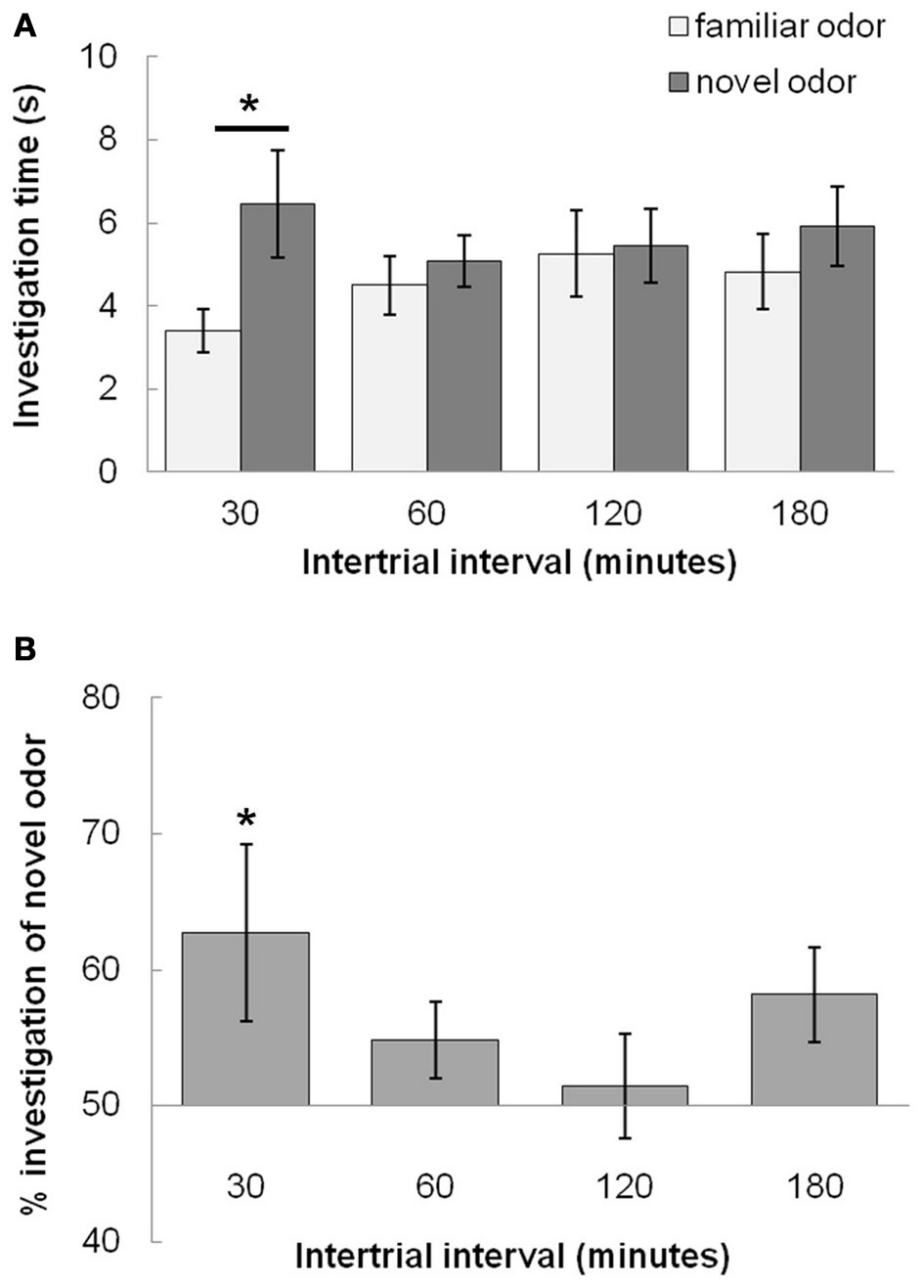
**Odor recognition memory duration (Experiment 1). (A)** The graph shows average investigation times for familiar and novel odors during *Trial 2* for 30, 60, 120, and 180 min ITIs. Rats investigate the novel odor more than the familiar odor only at the 30 min ITI, but not at longer ITIs (60, 120, or 180 min ITIs). An ^*^ indicates significant values for pairwise comparisons (*p* < 0.05) for investigation time between novel and familiar odors (Fisher's LSD tests). **(B)** The graph shows the relative investigation of the novel odor as compared to the familiar odor by showing percentage of total investigation time in which rats investigate the novel odor; this is a measure for odor recognition memory.

In Experiment 1, we tested the duration of rats' memory using an odor recognition task. Figure 3 shows the average investigation time of the novel and familiar odor (Figure 3A) and the percentage of investigation spent dedicated to the novel odor (Figure 3B), both during *Trial 2* and averaged across rats (*n* = 12). These results indicate that rats exhibit a robust novelty response following an ITI of 30 min that is largely diminished by 60 min. A MANOVA revealed a significant effect on investigation time between novel and familiar odorants during the test trial [*F*_(1, 41)_ = 20.06; *p* < 0.001] as well as a significant interaction between investigation time and ITI [*F*_(3, 41)_ = 7.416; *p* < 0.001] indicating that the difference in investigation time was dependent on ITI. *Posthoc* tests (Fisher LSD) show that only rats tested at 30 min ITI investigated the novel odor significantly more than the familiar odor in the second trial (*p* < 0.05).

### Bright light, sound and bulbar NE modulate odor memory (experiment 2)

Having established the time course of odor recognition memory under control conditions, we examined whether an acute mild stressor or manipulation of bulbar NE during the familiarization trial (*Trial 1*) could modulate odor memory. We tested if memory could be enhanced by these manipulations by using ITIs long enough for control rats to no longer investigate the novel more than the familiar odor (60 and 180 min). We then tested if these manipulations could decrease odor memory duration by using an ITI at which control rats could remember the odor (30 min).

**Figure 4:**
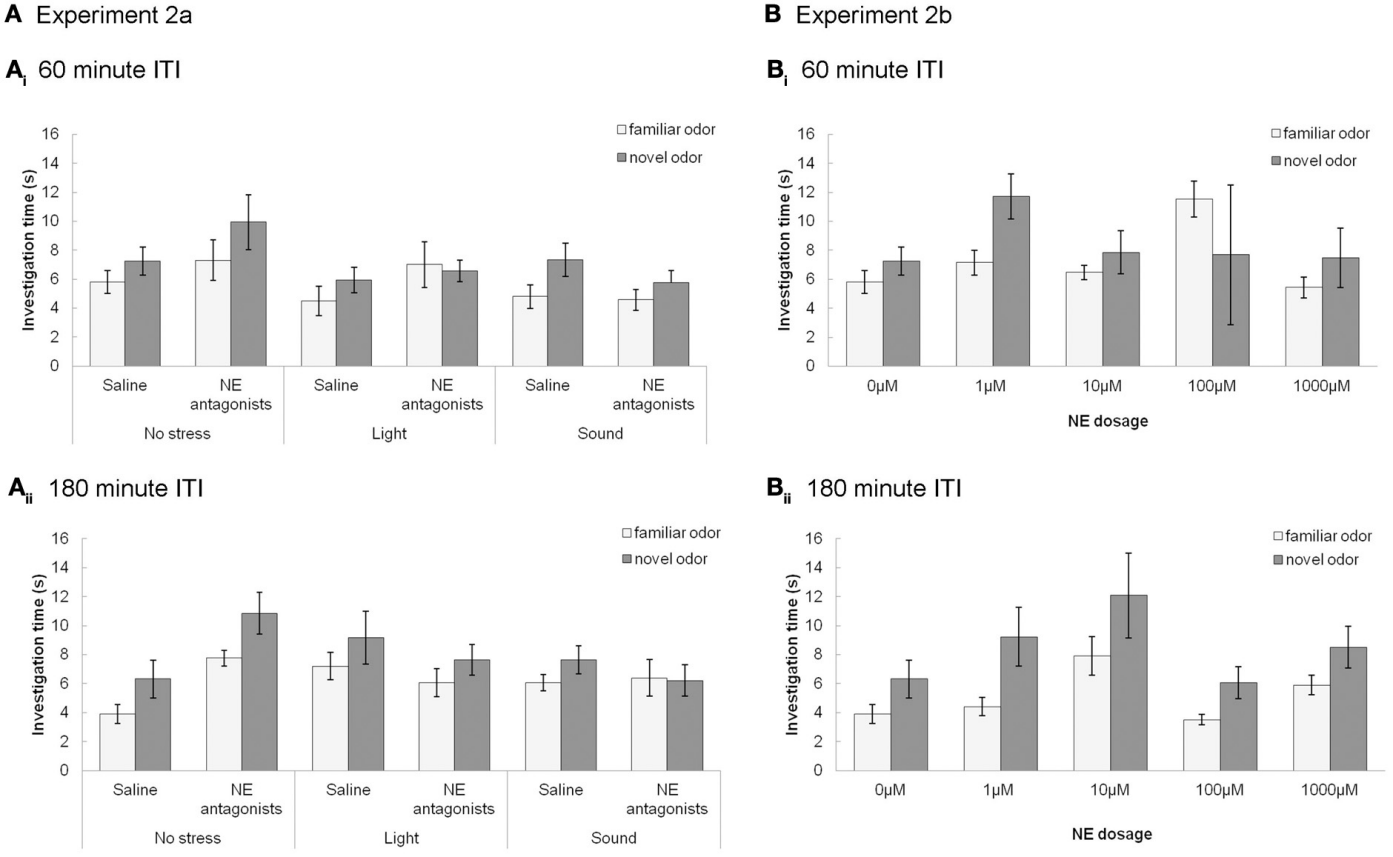
**NE and stressors have no effect at longer, 60 and 180 min, ITIs. (A)** Experiments 2a **(A)** and 2b **(B)**: The graphs show the average investigation times to familiar and novel odorants during trials 1&2 at 60 **(A)** or 180 **(B)** min ITI, as a function of stressors **(Ai)** and **(Bi)** or NE dosage **(Aii)** and **(Bii)**. At these ITIs no treatment groups investigate the novel odor significantly more than the familiar odor during *Trial 2*. Stressors and infusion of NE antagonist cocktail have no effect.

At ITIs longer than 30 min (60 and 180 min), the control group (saline, no stress) do not investigate the novel odor more than the familiar odor (similar to **Experiment 1** in Figure 3), and stress and drug do not have an effect on odor memory (Figure 4). For **Experiment 2a** (Figure 4A), at ITIs of 60 and 180, although MANOVAs reveal a significant overall effect of investigation time between the familiar and novel odor in *Trial 2* when data is pooled across all experimental groups [Wilk's Lambda: 60 min: *F*_(1, 57)_ = 10.306; *p* = 0.002; 180 min: *F*_(1, 49)_ = 7.442; *p* = 0.009] there is no interaction of investigation time with stress [60 min: *F*_(2, 57)_ = 2.023; *p* = 0.142; 180 min: *F*_(2, 49)_ = 0.649; *p* = 0.527] or drug [saline or NE antagonist cocktail; 60min: *F*_(1, 57)_ = 0.001; *p* = 0.974; 180 min: *F*_(1, 49)_ = 0.168; *p* = 0.684]. At these two ITIs, no treatment group investigated the novel odor significantly more than the familiar odor (*p* > 0.05 in all cases). For **Experiment 2b** (Figure 4B), a MANOVA with level of NE (0, 1, 10, 100, or 1000 μM) as a between subjects effect showed no effect of investigation time overall at 60 min [*F*_(1, 47)_ = 2.162; *p* = 0.148], but did show a significant effect of investigation time at 180 min [*F*_(1, 41)_ = 16.912; *p* < 0.001] but no interaction between drug and investigation time [*F*_(4, 41)_ = 0.370; *p* = 0.823]. No individual treatment group investigated the novel odor significantly more than the familiar odor during *Trial 2* (*p* > 0.05 in all cases).

**Figure 5:**
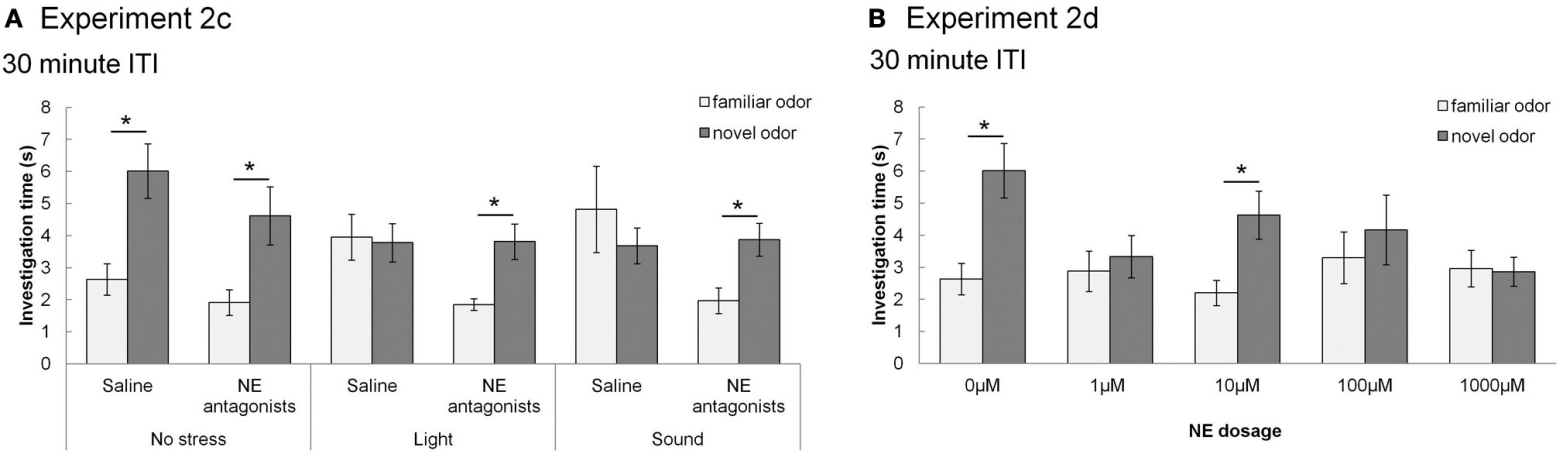
**NE and stressors suppress memory at 30 min ITIs (Experiment 2c and 2d)**. These graph show average investigation times to familiar and novel odors in trials 1&2 under different experimental conditions with an ITI 30 min. ^*^ indicates a significant difference in investigation time between novel and familiar odor in *Trial 2*. **(A) Experiment 2c**: In non-stressed conditions (either with saline or NE antagonists cocktail infusion into the olfactory bulb prior to the familiarization trial), rats investigate the novel odor significantly more than the familiar odor. However, when either light or sound is implemented during the familiarization trial, rats do not investigate the novel odor more than the familiar odor. The effect of light or sound is counteracted by infusion of NE antagonist cocktail before the familiarization trial. **(B) Experiment 2d**: Saline infused control rats and rats infused with 10 μM NE investigate the novel odor significantly more than the familiar odor during *Trial 2*.

In contrast, Figure 5 shows that with subsequent testing at a shorter, 30 min ITI, control rats were able to detect the novel odorant indicating memory for the familiar odor (similar to Figures 3, 5). For **Experiment 2c** (Figure 5A), with 30 min ITIs, a MANOVA reveals a significant effect of investigation time between familiar and novel odor during *Trial 2* [*F*_(1, 45)_ = 11.083; *p* = 0.002]. However, unlike the longer ITIs, there was a significant interaction with stress [*F*_(2, 45)_ = 4.765; *p* = 0.013] but not drug [saline, NE antagonist cocktail; *F*_(1, 45)_ = 2.611; *p* = 0.113]. Control rats (no stress + saline) displayed significantly higher investigation of novel compared to familiar odors (*p* < 0.05), as did rats infused with NE antagonists before stressors were applied (*p* < 0.05). Rats stressed with light or sound did not show significant differences in novel vs. familiar odor investigation. (Figure 5B).

For **Experiment 2d** (Figure 5B), there was an overall effect of investigation time when NE levels (0, 1, 10, 100, 1000 μM NE) were analyzed [*F*_(1, 41)_ = 11.094, *p* = 0.002], as well as significant interaction between investigation time and NE levels [*F*_(4, 41)_ = 3.2334; *p* = 0.042]. Rats infused with 1, 100, and 1000 μM NE before the familiarization trial did not investigate the novel odor more than the familiar odor (*p* > 0.05), whereas those infused with 10 μM NE did (*p* < 0.05) (Figure 5B).

**Figure 6:**
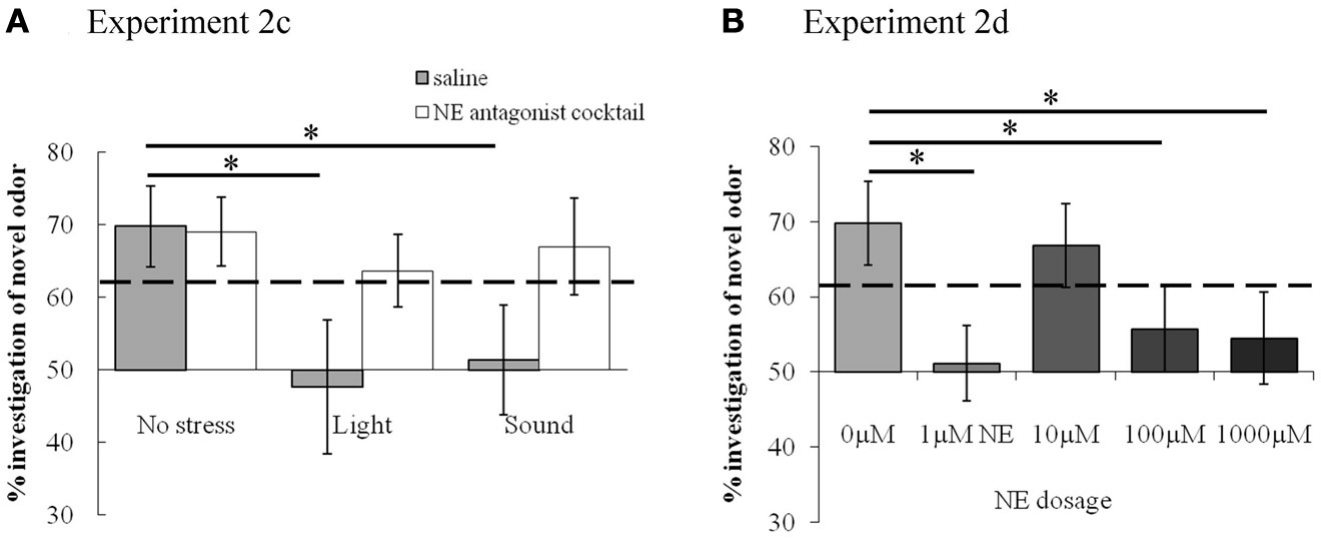
**Summary of effects of stress and NE on short term odor memory**. Light and sound stressors, as well as 1 μM NE, reduce the relative amount of investigation of the novel odor. The graph shows relative investigation times of the novel odorant during *Trial 2* (test trial) with stressor **(A)** or NE infusions **(B)**. The dashed line indicates the relative time above which the difference between novel and familiar odor investigation was significant, as analyzed from raw data and detailed in results section. ^*^indicates significant differences between treatment groups.

In summary, the experiments using the 30 min ITI show that memory for the odor, present in saline infused control conditions, is impaired by light or sound stress as well as by NE infusions. Blockade of NE receptors during odor encoding prevents the effects of light and sound stress. The relative investigation of the novel odor as compared to total investigation during the memory trial is commonly used as a measure for the strengths of odor recognition memory (Petrulis and Johnston, 1999; Johnston and Peng, 2000; Veyrac et al., 2007; Guerin et al., 2008). To compare the relative investigation of the novel odor across experimental conditions in the 30 min ITI experiment, we used an ANOVA with experimental group as the main effect and relative investigation time as the dependent variable. Statistical analysis showed a significant effect of treatment group [*F*_(9, 81)_ = 3.309; *p* = 0.002], with light and sound treated saline rats, 1 μM, 100 μM and 1000 μM NE infused rats investigating significantly different from saline treated non stressed rats (*p* < 0.01 in all cases). Figure 6 shows the summary of relative investigation times across the treatment groups used in experiments 2c and 2d (30 min ITI). This summary clearly shows that (a) light and sound stress during encoding impair memory at 30 min in a manner similar to a range of NE dosages, and (b) that blockade of NE receptors counteract the effect of light and sound, suggesting a noradrenergic contribution in the OB to the effects of light and sound.

### Control experiments for activity patterns during trial 1 of experiment 2

**Figure 7:**
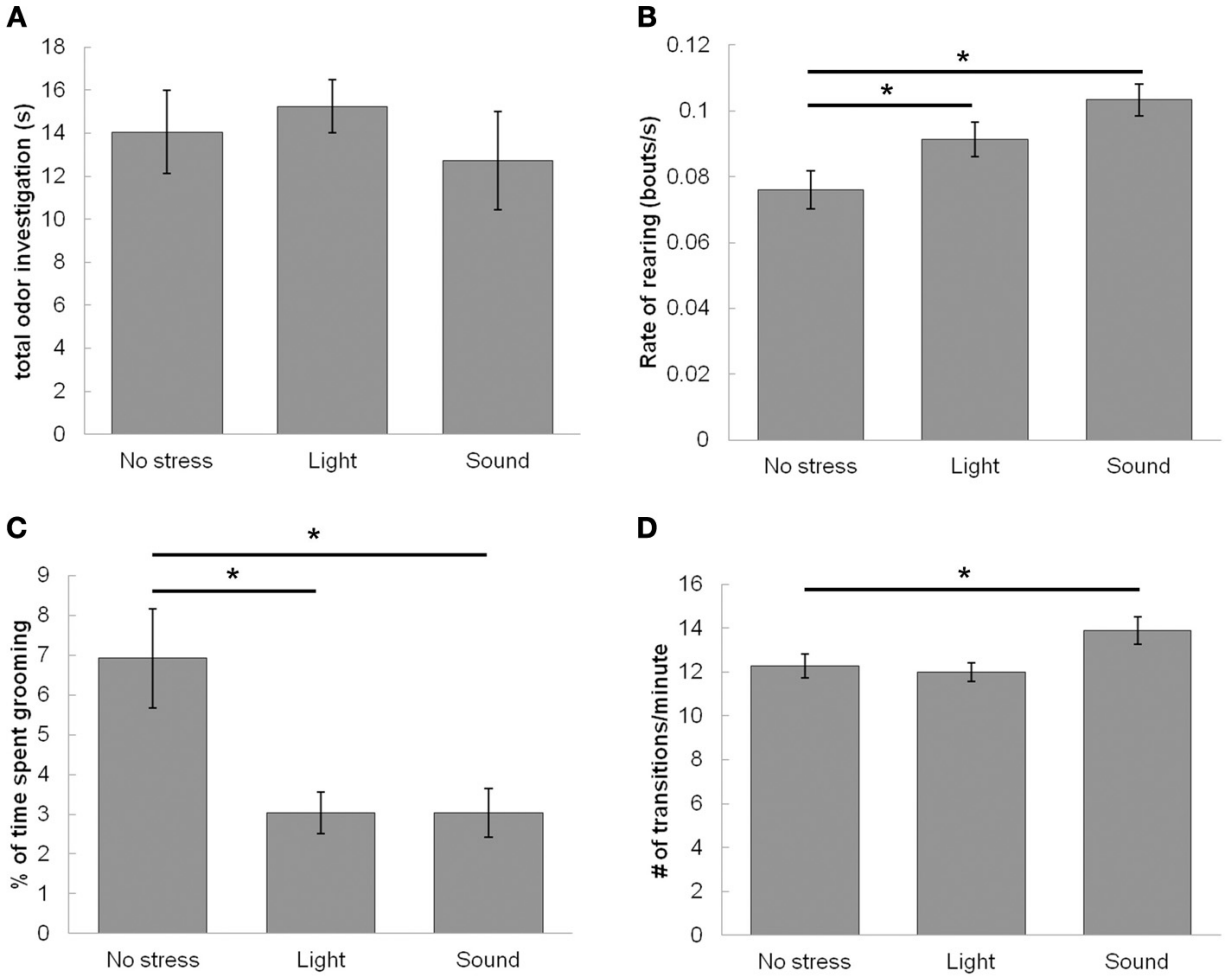
**Effect of stressors on locomotor activity during the familiarization trial (*Trial 1*)**. Stressors enhance exploratory and suppress resting-state activities of rats during the familiarization trial of the odor recognition task while leaving investigation of odor unaltered. **(A)** The graph shows that neither stressor has any effect on total time of odor investigation during the familiarization trial when compared to the non stressed condition. **(B)** The graph shows the number of rearing events per second, suggesting that stressors (both light and sound) enhance the rate of non-odor based rearing events. **(C)** The graph shows that stressors (both light and sound) suppress percentage of time spent grooming. **(D)** The graph shows that sound but not light increases the rate of transitioning between quadrants of the testing chamber compared to the non-stressed condition. ^*^indicates significant pairwise comparisons.

Figure 7A shows the results of testing if the effects seen above could be attributed to a change in duration of investigation during the familiarization trial. To do so, we tested the effects of stress (no stress, bright light, sound) and drug (saline, NE antagonist cocktail) on total investigation time during the familiarization trial. ANOVA with stress (no stress, light or sound) or drug (saline or NE blocker) showed no effect of either stress [*F*_(2, 157)_ = 1.488; *p* = 0.220] or drug [*F*_(1, 157)_ = 0.631]. Additionally, to test if NE dosage affected investigation, we ran an ANOVA with NE dosage (0, 10, 100, 1000 μM NE) on investigation time in the familiarization trial [*F*_(4, 135)_ = 1.848; *p* = 0.123]. This suggests results were not due to changes in investigation of the odor during familiarization.

To further investigate how stressors and drugs affect behavior during the familiarization trial, we measured a variety of locomotor/activity measures during the familiarization trial (Figures 7B–D). First, we found the rate of rearing during a trial, not including bouts where the animal reared to investigate the odors. ANOVA results show a significant effect of stress on the rate of rearing during the familiarization trial [*F*_(2, 159)_ = 6.51, *p* = 0.0019]. Although a trend exists for both bright light and sound increasing rate of rearing, *posthoc* analysis shows a significant increase as compared to saline infused control rats only with sound (Figure 7B). There is no interaction between stress and drug [saline or NE antagonist cocktail; *F*_(1, 159)_ = 0.44, *p* = 0.51]. There was also no effect of any drug treatment (saline, 1, 10, 100, 1000 μM NE, NE antagonists) on rearing [*F*_(5, 150)_ = 0.045, *p* = 1.0].

Next, we measured amount of time grooming during the trial, and normalized the value to length of video analyzed (Figure 7C). ANOVA results show a significant effect of stress (no stress, light, sound) on percentage of time spent grooming [*F*_(2, 157)_ = 6.921, *p* = 0.001], but no effect of drug (saline or NE antagonist) [*F*_(1, 157)_ = 0.005, *p* = 0.94], or interaction between stress and drug [saline or NE antagonist cocktail; *F*_(1, 159)_ = 0.124, *p* = 0.88]. *Posthocs* (Fisher's LSD) show that bright light and sound both decrease the percentage of time rats spend grooming when compared to no stress control sessions (Figure 7C). There was also no effect of NE dosage (saline, 1, 10, 100, 1000 μM NE, NE antagonists) on grooming [*F*_(5, 150)_ = 0.05, *p* = 1.0].

Finally, we measured locomotor activity throughout the trial by dividing the chamber into four quadrants and measuring the rate of crossings within these quadrants (quadrants shown in Figures 1, 7D). There was a main effect of stress on rate of transitioning throughout the chamber [*F*_(2, 157)_ = 3.73, *p* = 0.026], whereby *posthoc* LSD tests show that sound but not bright light increase rate of transitions throughout the test chamber when compared to no stress controls (Figure 7D). However, there was no effect of drug [saline or NE blockers, *F*_(2, 157)_ = 0.17, *p* = 0.90], and no interaction between drug and stress [*F*_(2, 157)_ = 1.058, *p* = 0.35]. Additionally there was no effect of any drug treatment (saline, 1, 10, 100, 1000 μM NE, NE antagonists) on rate of transitions [*F*_(5, 150)_ = 0.87, *p* = 0.51].

## Discussion

We show here that moderate and acute stressors during acquisition of an odor memory can later suppress that memory. The effect of stressors is impaired by local bulbar blockade of noradrenergic processing and can be mimicked by local infusion of NE (Figures 5, 6). This suggests that NE release from the LC in response to the stressors during memory acquisition (Pavcovich and Ramirez, 1991; Rajkowski et al., 1994; Sands et al., 2000; Valentino and Van Bockstaele, 2008) modulates OB processing to suppress acquisition or expression of the odor memory. Future studies are necessary to determine the particular origin of this effect; whether it abolishes encoding of the memory altogether or alters duration of the odor memory. Regardless of these options, however, these results suggest an importance of state-dependent processing of non-social odors, in this case produced by stressors and dependent upon noradrenergic inputs to the OB.

Our results are in agreement with studies in other brain systems. Chronic stress, for instance, is associated with elevated NE levels and has been linked to suppression of object recognition memory in male rats (Beck and Luine, 1999; Bowman et al., 2003). Additionally, acute stress both during and after acquisition of an object or spatial memory tends to suppress later memory expression (Cazakoff et al., 2010). Although these results are complementary to present findings, it is important to note that odor recognition memory (Cleland and Sethupathy, 2006; Wilson and Sullivan, 2011); involves circuitry and specific plasticity that may not be involved in spatial and object recognition memory (DeVito and Eichenbaum, 2010; Izquierdo et al., 2002), and vice versa. In other cases, NE can enhance memory (Roozendaal et al., 2008), highlighting the notion that NE can produce differing results dependent upon task, dosage, brain area, and timing-dependent effects.

We found that in our behavioral paradigm light and sound stressors alter overall activity levels during the odor acquisition trial when the animal would be stressed (Figure 7). Rats were more likely to engage in rearing while being less likely to groom. Additionally, sound but not light enhanced rate of transitions throughout the chamber. Overall, these results suggest that, similar to previous studies (Archer, 1973; Roth and Katz, 1979; Katz et al., 1981), light and sound tend to increase active exploration while inhibiting passive activities such as grooming. These active states have often been shown to relate to higher levels of NE levels in downstream targets of the LC (Pavcovich and Ramirez, 1991; Rajkowski et al., 1994; Sands et al., 2000; Valentino and Van Bockstaele, 2008). Unsurprisingly, the effects we find on exploratory activities are maintained regardless of whether the animal is given bulbar infusions of vehicle or NE antagonists, and are not altered by infusions of any dosage of NE. This maintenance of effect confirms that modulation of other brain areas than the OB promotes these locomotor effects.

With respect to olfactory memory, local blockade of NE receptors in the OB only was sufficient to block the suppression of memory due to stressors; suggesting that the OB at least partially mediates a form of plasticity underlying odor recognition memory (as suggested by the Shea et al., 2008 study). We have previously shown in mice and rats that the formation of a non-associative olfactory memory can be suppressed by bulbar manipulations (Guerin et al., 2008; McNamara et al., 2008; Chaudhury et al., 2010). In mice and rats, bulbar blockade of NMDA receptors impaired the formation of a non-associative memory (McNamara et al., 2008; Chaudhury et al., 2010) and in rats we showed that the associated changes in mitral cell firing were also impaired by local blockade of NMDA receptors (Chaudhury et al., 2010). In mice, lesions of noradrenergic neurons in the LC prevented the formation of odor memory; the effect of these non-specific noradrenergic lesions on olfactory memory formation could be restored by local bulbar infusions of NE (Guerin et al., 2008).

The suppression of memory duration by bulbar NE or stressor is one possible interpretation of our results. Based on data from other groups, alternative interpretations need to be considered. OB NE could act to reinforce the presence of the familiar odor, rendering it more attractive and therefore leading to higher investigation times in a subsequent trial. This type of preference modulation through NE/stressor mechanisms has been shown extensively in neonatal rodents (Moriceau and Sullivan, 2004; Moriceau et al., 2009, 2010). On the other hand, extensive results from our own group using a classical habituation paradigm with repeated odor presentations in the presence of bulbar NE have not shown such a change in odor preference as in these studies habituation itself was not modulated by NE (Wilson and Sullivan, 1992; Wilson and Stevenson, 2003; Veyrac et al., 2007; Guerin et al., 2008; Mandairon et al., 2008; Mandairon and Linster, 2009; Escanilla et al., 2010; Freedman et al., 2013).

Previous data from our group has shown a general enhancement of odor detection and discrimination at very low odor concentration by bulbar NE (Escanilla et al., 2010), which may seem contradictory to the results presented here. This apparent disconnect could potentially be explained by the time course of these behaviors and potentially different roots in the underlying plasticity. The 2010 study showed effects immediately following habituation, while we tested at 30 min and beyond following habituation. In that light, our results may indicate a more interesting tradeoff between an immediate enhancement of detection and discrimination and subsequent suppression of short term memory of that same odor.

Stress, arousal, and NE generally produce an inverted U-shaped curve that predicts performance on memory tasks (Joels et al., 2006; Sandi and Pinelo-Nava, 2007), also seen in recordings of NE modulation in OB slices (Nai et al., 2009) as well as olfactory mediated behaviors (Escanilla et al., 2010). In the present study, the effect of mild stressors could be said to be mimicked by a low dose of NE (1μM NE, Figures 5, 6). The response to NE then follows a non-linear, dose-response curve reminiscent of the typical inverted U-shaped dose response curve of lower and higher dosages suppressing memory performance more so than intermediate dosages (Figures 5B, 6). This result is also similar to the curve describing effects of NE on mitral cell inhibition triggered by NE infusions in OB slices (Nai et al., 2009).

Overall, our results strongly suggest modulation of bulbar processing during memory acquisition by stress-induced NE release into the OB. Using stressors is a valuable, physiologically and behaviorally relevant mechanism to manipulate NE levels behaviorally rather than by infusion or stimulation.

## Author contributions

Laura C. Manella was the primary author, responsible for designing experiments, collecting, recording, analyzing data, and writing the manuscript. Samuel Alperin collected and recorded data. Christiane Linster is the corresponding author and the principle investigator responsible for guiding this research and aiding and advising in analysis of data and writing the manuscript.

### Conflict of interest statement

The authors declare that the research was conducted in the absence of any commercial or financial relationships that could be construed as a potential conflict of interest.

## References

[B2] Archer J. (1973). Tests for emotionality in rats and mice: a review. Anim. Behav. 21, 205–235 10.1016/S0003-3472(73)80065-X 4578750

[B4] Aston-Jones G. Rajkowski J. Cohen J. Uylings H. B. M. van Eden G. G. de Bruin J. P. C. (2000). Locus coeruleus and regulation of behavioral flexibility and attention. Prog. Brain Res. 126, 165–182 10.1016/S0079-6123(00)26013-5 11105646

[B5] Aston-Jones G. Rajkowski J. Kubiak P. Valentino R. J. Shipley M. T. Holstege R. B. G. (1996). Chapter 23 Role of the locus coeruleus in emotional activation. Prog. Brain Res. 107, 379–402 10.1016/S0079-6123(08)61877-4 8782532

[B6] Axelrod J. Reisine T. D. (1984). Stress hormones: their interaction and regulation. Science 224, 452–459 10.1126/science.6143403 6143403

[B7] Beck K. D. Luine V. N. (1999). Food deprivation modulates chronic stress effects on object recognition in male rats: role of monoamines and amino acids. Brain Res. 830, 56–71 10.1016/S0006-8993(99)01380-3 10350560

[B8] Bevins R. A. Besheer J. (2006). Object recognition in rats and mice: a one-trial non-matching-to-sample learning task to study recognition memory. Nat. Protoc. 1, 1306–1311 10.1038/nprot.2006.205 17406415

[B9] Bowman R. E. Beck K. D. Luine V. N. (2003). Chronic stress effects on memory: sex differences in performance and monoaminergic activity. Horm. Behav. 43, 48–59 10.1016/S0018-506X(02)00022-3 12614634

[B10] Cazakoff B. N. Johnson K. J. Howland J. G. (2010). Converging effects of acute stress on spatial and recognition memory in rodents: a review of recent behavioural and pharmacological findings. Prog. Neuropsychopharmacol. Biol. Psychiatry 34, 733–741 10.1016/j.pnpbp.2010.04.002 20394792

[B11] Chaudhury D. Manella L. Arellanos A. Escanilla O. Cleland T. A. Linster C. (2010). Olfactory bulb habituation to odor stimuli. Behav. Neurosci. 124, 490–499 10.1037/a0020293 20695648 PMC2919830

[B12] Cleland T. A. Sethupathy P. (2006). Non-topographical contrast enhancement in the olfactory bulb. BMC Neurosci. 7:7 10.1186/1471-2202-7-7 16433921 PMC1368991

[B31] DeVito L. M. Eichenbaum H. (2010). Distinct contributions of the hippocampus and medial prefrontal cortex to the what-where-when components of episodic-like memory in mice. Behav. Brain Res. 215, 318–325 10.1016/j.bbr.2009.09.014 19766146 PMC2891645

[B14] Devore S. Linster C. (2012). Noradrenergic and cholinergic modulation of olfactory bulb sensory processing. Front. Behav. Neurosci. 6:52 10.3389/fnbeh.2012.00052 22905025 PMC3417301

[B14a] Doucette W. Milder J. Restrepo D. (2007). Adrenergic modulation of olfactory bulb circuitry affects odor discrimination. Learn Mem. 14, 539–547 10.1101/lm.606407 17686948 PMC1951793

[B15] Escanilla O. Arrellanos A. Karnow A. Ennis M. Linster C. (2010). Noradrenergic modulation of behavioral odor detection and discrimination thresholds in the olfactory bulb. Eur. J. Neurosci. 32, 458–468 10.1111/j.1460-9568.2010.07297.x 20618829

[B16] Ferreira G. Gervais R. Durkin T. P. Lévy F. (1999). Postacquisition scopolamine treatments reveal the time course for the formation of lamb odor recognition memory in parturient ewes. Behav. Neurosci. 113, 136–142 10.1037/0735-7044.113.1.136 10197913

[B17] Freedman K. G. Radhakrishna S. Escanilla O. Linster C. (2013). Duration and specificity of olfactory nonassociative memory. Chem. Senses 38, 369–375 10.1093/chemse/bjt010 23513053

[B18] Guerin D. Peace S. T. Didier A. Linster C. Cleland T. A. (2008). Noradrenergic Neuromodulation in the olfactory bulb modulates odor habituation and spontaneous discrimination. Behav. Neurosci. 122, 816–826 10.1037/a0012522 18729635 PMC2562590

[B28] Izquierdo L. A. Barros D. M. Vianna M. R. M. Coitinho A. deDavid e Silva T. Choi H. (2002). Molecular pharmacological dissection of short- and long-term memory. Cell. Mol. Neurobiol. 22, 269–287 10.1023/A:1020715800956 12469870 PMC11533753

[B19] Jiang M. Griff E. R. Ennis M. Zimmer L. A. Shipley M. T. (1996). Activation of locus coeruleus enhances the responses of olfactory bulb mitral cells to weak olfactory nerve input. J. Neurosci. 16, 6319–6329 8815911 10.1523/JNEUROSCI.16-19-06319.1996 PMC6579166

[B20] Joels M. Pu Z. Wiegert O. Oitzl M. S. Krugers H. J. (2006). Learning under stress: how does it work? Trends Cogn. Neurosci. 10, 152–158 10.1016/j.tics.2006.02.002 16513410

[B21] Johnston R. E. Peng M. (2000). The vomeronasal organ is involved in discrimination of individual odors by males but not by females in golden hamsters. Physiol. Behav. 70, 537–549 10.1016/S0031-9384(00)00304-8 11111009

[B25] Jurado-Berbel P. Costa-Miserachs D. Torras-Garcia M. Coll-Andreu M. Portell-Cortés I. (2010). Standard object recognition memory and “what” and “where” components: improvement by post-training epinephrine in highly habituated rats. Behav. Brain Res. 207, 44–50 10.1016/j.bbr.2009.09.036 19788899

[B26] Katz R. J. Roth K. A. Carroll B. J. (1981). Acute and chronic stress effects on open field activity in the rat: Implications for a model of depression. Neurosci. Biobehav. Rev. 5, 247–251 10.1016/0149-7634(81)90005-1 7196554

[B27] Koyama Y. Jodo E. Kayama Y. (1994). Sensory responsiveness of “Broad-spike” neurons in the laterodorsal tegmental nucleus, locus coeruleus and dorsal raphe of awake rats: Implications for cholinergic and monoaminergic neuron-specific responses. Neuroscience 63, 1021–1031 10.1016/0306-4522(94)90569-X 7700507

[B29] Linster C. Menon A. V. Singh C. Y. Wilson D. A. (2009). Odor-specific habituation arises from interaction of afferent synaptic adaptation and intrinsic synaptic potentiation in olfactory cortex. Learn. Mem. 16, 452–459 10.1101/lm.1403509 19553383 PMC3263734

[B30] Linster C. Nai Q. Ennis M. (2011). Nonlinear effects of noradrenergic modulation of olfactory bulb function in adult rodents. J. Neurophysiol. 105, 1432–1443 10.1152/jn.00960.2010 21273323 PMC3075300

[B40] Mandairon N. Linster C. (2009). Odor perception and olfactory bulb plasticity in adult mammals. J. Neurophysiol. 101, 2204–2209 10.1152/jn.00076.2009 19261715

[B33] Mandairon N. Peace S. Karnow A. Kim J. Ennis M. Linster C. (2008). Noradrenergic modulation in the olfactory bulb influences spontaneous and reward-motivated discrimination, but not the formation of habituation memory. Eur. J. Neurosci. 27, 1210–1219 10.1111/j.1460-9568.2008.06101.x 18364038

[B34] Mandairon N. Stack C. Kiselycznyk C. Linster C. (2006). Broad activation of the olfactory bulb produces long lasting changes in odor perception. Proc. Natl. Acad. Sci. U.S.A. 103, 13543–13548 10.1073/pnas.0602750103 16938883 PMC1569199

[B36] McNamara A. M. Magidson P. D. Linster C. Wilson D. A. Cleland T. A. (2008). Distinct neural mechanisms mediate olfactory memory formation at different timescales. Learn. Mem. 15, 117–125 10.1101/lm.785608 18299438 PMC2275653

[B38a] Moriceau S. Roth T. L. Sullivan R. M. (2010). Rodent model of infant attachment learning and stress. Dev. Psychobiol. 52, 651–660 10.1002/dev.20482 20730787 PMC4334117

[B37] Moriceau S. Shionoya K. Jakubs K. Sullivan R. M. (2009). Early-life stress disrupts attachment learning: The role of amygdala corticosterone, locus ceruleus corticotropin releasing hormone, and olfactory bulb norepinephrine. J. Neurosci. 29, 15745–15755 10.1523/JNEUROSCI.4106-09.2009 20016090 PMC3345266

[B38] Moriceau S. Sullivan R. M. (2004). Corticosterone influences on mammalian neonatal sensitive-period learning. Behav. Neurosci. 118, 274–281 10.1037/0735-7044.118.2.274 15113251 PMC1868531

[B39] Nai Q. Dong H.-W. Hayar A. Linster C. Ennis M. (2009). Noradrenergic regulation of GABAergic inhibition of main olfactory bulb mitral cells varies as a function of concentration and receptor subtype. J. Neurophysiol. 101, 2472–2484 10.1152/jn.91187.2008 19279145 PMC2681435

[B41] Okuda S. Roozendaal B. McGaugh J. L. (2004). Glucocorticoid effects on object recognition memory require training-associated emotional arousal. Proc. Natl. Acad. Sci. U.S.A. 101, 853–858 10.1073/pnas.0307803100 14711996 PMC321770

[B42] Pavcovich L. A. Ramirez O. A. (1991). Time course effects of uncontrollable stress in locus coeruleus neuronal activity. Brain Res. Bull. 26, 17–21 10.1016/0361-9230(91)90186-N 2015515

[B43] Petrulis A. Johnston R. E. (1999). Lesions centered on the medial amygdala impair scent-marking and sex-odor recognition but spare discrimination of individual odors in female golden hamsters. Behav. Neurosci. 113, 345–357 10.1037/0735-7044.113.2.345 10357459

[B44] Rajkowski J. Kubiak P. Aston-Jones G. (1994). Locus coeruleus activity in monkey: Phasic and tonic changes are associated with altered vigilance. Brain Res. Bull 35, 607–616 10.1016/0361-9230(94)90175-9 7859118

[B45] Robert E. J. (1993). Memory for individual scent in hamsters (*Mesocricetus auratus*) as assessed by habituation methods. J. Comp. Psychol. 107, 201–207 10.1037/0735-7036.107.2.201 8370274

[B46] Roozendaal B. Castello N. A. Vedana G. Barsegyan A. McGaugh J. L. (2008). Noradrenergic activation of the basolateral amygdala modulates consolidation of object recognition memory. Neurobiol. Learn. Mem. 90, 576–579 10.1016/j.nlm.2008.06.010 18657626 PMC2572617

[B47] Roth K. A. Katz R. J. (1979). Stress, behavioral arousal, and open field activity–a reexamination of emotionality in the rat. Neurosci. Biobehav. Rev. 3, 247–263 10.1016/0149-7634(79)90012-5 542239

[B13] Sandi C. Pinelo-Nava M. T. (2007). Stress and memory: behavioral effects and neurobiological mechanisms. Neural Plast. 2007:78970 10.1155/2007/78970 18060012 PMC1950232

[B48] Sands S. A. Strong R. Corbitt J. Morilak D. A. (2000). Effects of acute restraint stress on tyrosine hydroxylase mRNA expression in locus coeruleus of wistar and wistar-kyoto rats. Mol. Brain Res. 75, 1–7 10.1016/S0169-328X(99)00255-7 10648882

[B1] Sara S. J. (2009). The locus coeruleus and noradrenergic modulation of cognition. Nat. Rev. Neurosci. 10, 211–223 10.1038/nrn2573 19190638

[B49] Scullion G. A. Kendall D. A. Sunter D. Marsden C. A. Pardon M. C. (2009). Central noradrenergic depletion by DSP-4 prevents stress-induced memory impairments in the object recognition task. Neuroscience 164, 415–423 10.1016/j.neuroscience.2009.08.046 19720115

[B50] Shea S. D. Katz L. C. Mooney R., R. C. (2008). Noradrenergic induction of odor-specific neural habituation and olfactory memories. J. Neurosci. 28, 10711–10719 10.1523/JNEUROSCI.3853-08.2008 18923046 PMC2588668

[B51] Smith R. S. Weitz C. J. Araneda R. C. (2009). Excitatory actions of noradrenaline and metabotropic glutamate receptor activation in granule cells of the accessory olfactory bulb. J. Neurophysiol. 102, 1103–1114 10.1152/jn.91093.2008 19474170 PMC2724365

[B52] Valentino R. J. Van Bockstaele E. (2008). Convergent regulation of locus coeruleus activity as an adaptive response to stress. Eur. J. Pharmacol. 583, 194–203 10.1016/j.ejphar.2007.11.062 18255055 PMC2349983

[B53] Veyrac A. Nguyen V. Marien M. Didier A. Jourdan F. (2007). Noradrenergic control of odor recognition in a nonassociative olfactory learning task in the mouse. Cold Spring Harb. Lab. Press 12, 847–854 10.1101/lm.708807 18086828 PMC2151022

[B54] Walker D. L. Davis M. (1997). Anxiogenic effects of high illumination levels assessed with the acoustic startle response in rats. Biol. Psychiatry 42, 461–471 10.1016/S0006-3223(96)00441-6 9285082

[B55] Wilson D. A. Linster C. (2008). Neurobiology of a simple memory. J. Neurophysiol. 100, 2–7 10.1152/jn.90479.2008 18463176

[B56] Wilson D. A. Stevenson R. J. (2003). Olfactory perceptual learning: the critical role of memory in odor discrimination. Neurosci. Biobehav. Rev. 27, 307–328 10.1016/S0149-7634(03)00050-2 12946684

[B57] Wilson D. A. Sullivan R. M. (1992). Blockade of mitral/tufted cell habituation to odors by association with reward: a preliminary note. Brain Res. 594, 143–145 10.1016/0006-8993(92)91039-H 1467934

[B58] Wilson D. A. Sullivan R. M. (2011). Cortical processing of odor objects. Neuron 72, 506–519 10.1016/j.neuron.2011.10.027 22099455 PMC3223720

